# The Danish High Risk and Resilience Study—VIA 11: Study Protocol for the First Follow-Up of the VIA 7 Cohort −522 Children Born to Parents With Schizophrenia Spectrum Disorders or Bipolar Disorder and Controls Being Re-examined for the First Time at Age 11

**DOI:** 10.3389/fpsyt.2018.00661

**Published:** 2018-12-12

**Authors:** Anne A. E. Thorup, Nicoline Hemager, Anne Søndergaard, Maja Gregersen, Åsa Kremer Prøsch, Mette F. Krantz, Julie M. Brandt, Line Carmichael, Marianne Melau, Ditte V. Ellersgaard, Birgitte K. Burton, Aja N. Greve, Md Jamal Uddin, Jessica Ohland, Ayna B. Nejad, Line K. Johnsen, Anna Hester Ver Loren van Themaat, Anna K. Andreassen, Lotte Vedum, Christina B. Knudsen, Henriette Stadsgaard, Jens Richardt M. Jepsen, Hartwig Roman Siebner, Leif Østergaard, Vibeke F. Bliksted, Kerstin J. Plessen, Ole Mors, Merete Nordentoft

**Affiliations:** ^1^The Lundbeck Foundation Intiative for Integrative Psychiatric Research (iPSYCH), Aarhus, Denmark; ^2^Research Unit, Child and Adolescent Mental Health Center, University of Copenhagen, Copenhagen, Denmark; ^3^Mental Health Center Copenhagen, Research Unit, University of Copenhagen, Copenhagen, Denmark; ^4^Psychosis Research Unit, Aarhus University Hospital, Risskov, Denmark; ^5^Danish Research Centre for Magnetic Resonance, Section 714, Hvidovre Hospital, University of Copenhagen, Copenhagen, Denmark; ^6^Center for Neuropsychiatric Schizophrenia Research (CNSR) and Center for Clinical Intervention and Neuropsychiatric Schizophrenia Research, Mental Health Centre Glostrup, University of Copenhagen, Copenhagen, Denmark; ^7^Center of Functionally Integrative Neuroscience, Aarhus University, Department of Neuroradiology, Aarhus University Hospital, Aarhus, Denmark; ^8^Division of Adolescent and Child Psychiatry, Department of Psychiatry, Lausanne University Hospital, Lausanne, Switzerland

**Keywords:** parental schizophrenia, parental bipolar disorder, early signs of mental illness, longitudinal cohort, psychopathology, MR scanning

## Abstract

**Introduction:** Offspring of parents with severe mental illness have an increased risk of developing mental illnesses themselves. Familial high risk cohorts give a unique opportunity for studying the development over time, both the illness that the individual is predisposed for and any other diagnoses. These studies can also increase our knowledge of etiology of severe mental illness and provide knowledge about the underlying mechanisms before illness develops. Interventions targeting this group are often proposed due to the potential possibility of prevention, but evidence about timing and content is lacking.

**Method:** A large, representative cohort of 522 7-year old children born to parents with schizophrenia, bipolar disorder or controls was established based on Danish registers. A comprehensive baseline assessment including neurocognition, motor functioning, psychopathology, home environment, sociodemographic data, and genetic information was conducted from January 1, 2013 to January 31, 2016. This study is the first follow-up of the cohort, carried out when the children turn 11 years of age. By assessing the cohort at this age, we will evaluate the children twice before puberty. All instruments have been selected with a longitudinal perspective and most of them are identical to those used at inclusion into the study at age 7. A diagnostic interview, motor tests, and a large cognitive battery are conducted along with home visits and information from teachers. This time we examine the children's brains by magnetic resonance scans and electroencephalograms. Measures of physical activity and sleep are captured by a chip placed on the body, while we obtain biological assays by collecting blood samples from the children.

**Discussion:** Findings from the VIA 7 study revealed large variations across domains between children born to parents with schizophrenia, bipolar and controls, respectively. This study will further determine whether the children at familial risk reveal delayed developmental courses, but catch up at age 11, or whether the discrepancies between the groups have grown even larger. We will compare subgroups within each of the familial high risk groups in order to investigate aspects of resilience. Data on brain structure and physical parameters will add a neurobiological dimension to the study.

## Introduction

For decades, familial high-risk studies have shown that individuals who are born to parents with schizophrenia and affective disorders have a higher risk of developing mental disorders and neurocognitive impairments than non-predisposed individuals (1–3). Cohort studies of children with familial risk with assessments carried out over several time points in their lives allow us to study the early processes that precede illness manifestation. Such studies increase our understanding of the etiology of these complex diseases, and they give us an excellent starting point for developing a specialized and focused preventive approach in terms of early intervention for high risk individuals.

A meta-analysis concluded that ~55% of all familial high risk (FHR) children will experience some kind of mental illness during early adult life (1), and a third will have a severe mental illness (SMI). Offspring of parents with SMI have a higher risk of developing the same disorders as their parents, but also of developing other mental disorders (4). Former FHR studies have reported neuro-integrative problems, social impairments, deficits in attention, information processing and other neurocognitive functions, poorer neuromotor functions and early symptomatology such as anxiety, sleep and mood disorders (2, 3), among offspring of parents with SMI. Moreover, in the premorbid phase the early signs of later schizophrenia (SZ) or bipolar disorder (BD) can present themselves already in childhood as e.g., anxiety, depression, or other non-psychotic disorders (5, 6). The first results from The Danish High Risk and Resilience Study—VIA 7 confirmed this by showing that already by age seven, children born to parents diagnosed with SZ and BD present much higher rates of a psychiatric diagnosis as well as cognitive deficits (only FHR SZ) across several domains, and motor problems (7–10). However, research is sparse when it comes to the question whether these abnormalities or developmental delays diminish over time, indicating that the individuals only suffer from those deficits and symptoms in a transitory manner and then catch up compared with the typically developing children, or whether the problems become even worse. A comprehensive review (11) synthesizing the data of neurocognition in individuals with FHR of schizophrenia concluded on the basis of 30 studies and nine cognitive domains that mild cognitive deficits (i.e., intermediate/between healthy controls and individuals diagnosed with schizophrenia) are reliably present in young FHR individuals, and that longitudinal studies should aim to elucidate the trajectories of cognitive changes to improve early intervention strategies. Concerning individuals at FHR of bipolar disorder there is evidence that neurocognitive deficits also play a role (12), although studies are smaller and tests are seldom repeated at different ages. It is therefore relevant to carry out careful examinations of early signs or symptoms of mental disorders among high risk children several times before puberty to learn more about these individuals and the trajectories of their emerging symptoms or difficulties.

*Puberty* is a period characterized by massive changes in brain structures and connectivity as well as changes in physical appearance, hormonal status and psychological and social constitution (13, 14). It is also a period with high incidence rates for mental disorders and for some, it is a period with changes of behavior, including risk taking activities and new relational patterns, for example higher degrees of independency. From a developmental perspective it is a period in life that is of high importance, but also very complex to study since both age and hormonal status as well as social and psychological aspects matter when comparing individuals in e.g., a cohort. Social mechanisms like bullying or other forms of social defeat that take place in childhood and early adolescence are frequently reported and have systematically been shown to be directly related to later emergence of mental illnesses like depression (15) and other negative life outcomes. Attributional style or coping, however, have been less investigated and may be of importance.

*Brain changes* in schizophrenia are present in drug naïve adult patients, and the strongest risk factors exert their influence already in the pre- or perinatal period (16, 17). Lately, the study of endophenotypes and biomarkers has advanced the field of SZ and BD as well as our understanding of the neurodevelopmental nature of both disorders (18, 19). This approach will also allow for early identification and intervention of serious mental illness (20). Despite the fact that only populations of young individuals before the mean age for the onset of the disorder are genuinely “at high risk” (19), few studies to date have examined adolescents at risk with the same means of brain mapping, and none before the onset of puberty. Taken together, the existing literature suggests the study of familial high-risk individuals as fruitful for the understanding of correlates for vulnerability and resilience (compensatory mechanisms), but findings have been inconsistent. In particular, magnetic resonance scans (MR scans) before puberty will permit the study of brain changes during early disease formation and provide important information on differences between individuals with and without familial risk.

Patients with SZ and BD have a higher risk of *somatic comorbidity* than the background population, and they have higher mortality rates due to medical diseases and untreated physical conditions (21). It is likely that early markers of physical illness can be traced already in childhood, either because of common life style factors in the family and/or because of a shared genetic vulnerability to mental disorders and physical illnesses, but evidence is limited. It is therefore relevant to examine early markers of evolving physical illness in children with FHR for mental illness and to analyze whether variations can be explained solely by differences in life style factors such as nutrition, environment, sleep, and physical activity. Also, very little is known about whether e.g., frequency of absence from school and the children's health service use differ between the groups. A Danish register study demonstrated, however, that children with FHR for SMI are more likely not to complete elementary school or to score lower grades than children without a predisposition for mental illness (22).

*Family functioning* is very often influenced when one of the parents (or both) are suffering from a severe mental illness, also in cases when the child does not live with the affected parent, but only with the co-parent (23). The daily routines can suddenly be disrupted by behavior or needs due to the mentally ill parents and resources, both materially, socially, culturally, and emotionally may be scarce. Dysfunctional family patterns are often seen in several dimensions of family life such as problem solving, communication, role functioning, affective responsiveness, affective involvement and behavior control in families with parental mental illness (24). These dimensions are thoroughly described by the authors behind Family Assessment Device, a self-administrated and widely used questionnaire based on social and system theoretical principles to measure family functioning [FAD (25)].

Briefly, the first *results from the VIA 7 study* showed considerable associations between parental mental illness and several domains of child functioning. Children born to parents diagnosed with schizophrenia showed markedly impaired neurocognitive functioning on the majority of applied measures; i.e., processing speed, working memory, executive functioning, visuospatial functions, sustained attention, and declarative memory (7). At the same time, we found an increased risk of displaying mental health problems or even mental disorders among both groups of children with familial high risk, but in particular among the children with familial high-risk of schizophrenia. Of children with FHR for SZ 38.7% had a life time diagnosis, while this was found in 35.6% of the children with FHR for BD, and in 15.2% of the controls. Also dimensional measures of psychopathology showed this (8). Especially anxiety, ADHD, and stress and adjustment disorders were more prevalent in both of the FHR-groups. Children with FHR for SMI also showed impairments in motor functioning (9) and in sustained attention and interference control (10). Further, children with FHR for both mental disorders were found to be at a greater risk of growing up under low socioeconomic status and reported more adverse life events than the controls based on interviews with the caregivers (unpublished data). Results further indicate an increased risk for living in a home environment with insufficient levels of stimulation and support for children predisposed for SZ and to a lesser extent also BD, and also social cognition and language development was found to be impaired in the FHR-SZ group (manuscript in preparation).

Increasing our knowledge about both the risk and the protective factors that influence these children's development is important to improve our chances for identifying the most vulnerable groups of children. More knowledge concerning the underlying psychological and neurobiological mechanism will guide future developments of early interventions with a preventive approach.

## Aims

In a cohort of children with familial high risk for schizophrenia or bipolar disorder our aims are:

1. To improve insight into early disease processes in schizophrenia and bipolar affective disorder including symptom formation, impairments or delays of maturation in different domains of cognitive functioning incl. social cognition, paralleled by difference of brain structure and of patterns of brain activation compared to controls.
2. To investigate the development over time (i.e., from age 7 to age 11) of children with familial high risk compared to controls in the domains of neurocognition, psychopathology, social cognition, motor function and adverse life events and to identify and describe possible associations between familial high risk and factors that increase resilience in children.
3. To identify the influence of genetic and environmental exposures and their interactions.
4. To identify early amendable risk factors such as lack of stimulation and support, traumatic events during childhood, insufficient parenting, low socioeconomic status, neurocognitive and social cognitive deficits, and early, subtle signs of psychopathology.
5. To improve knowledge about physical health status among children with familial high risk for severe mental illness.

## Design

*The Danish High Risk and Resilience Study* is a representative nationwide cohort study consisting of 522 children born to parents with schizophrenia, bipolar disorder or neither of these disorders. The participating families were recruited from Danish registers and investigated thoroughly during 2013–2016 when the children were seven years old. The first assessment is referred to as the VIA 7 study, for details please see study protocol for the VIA 7 study (26).

**Figure 1:**
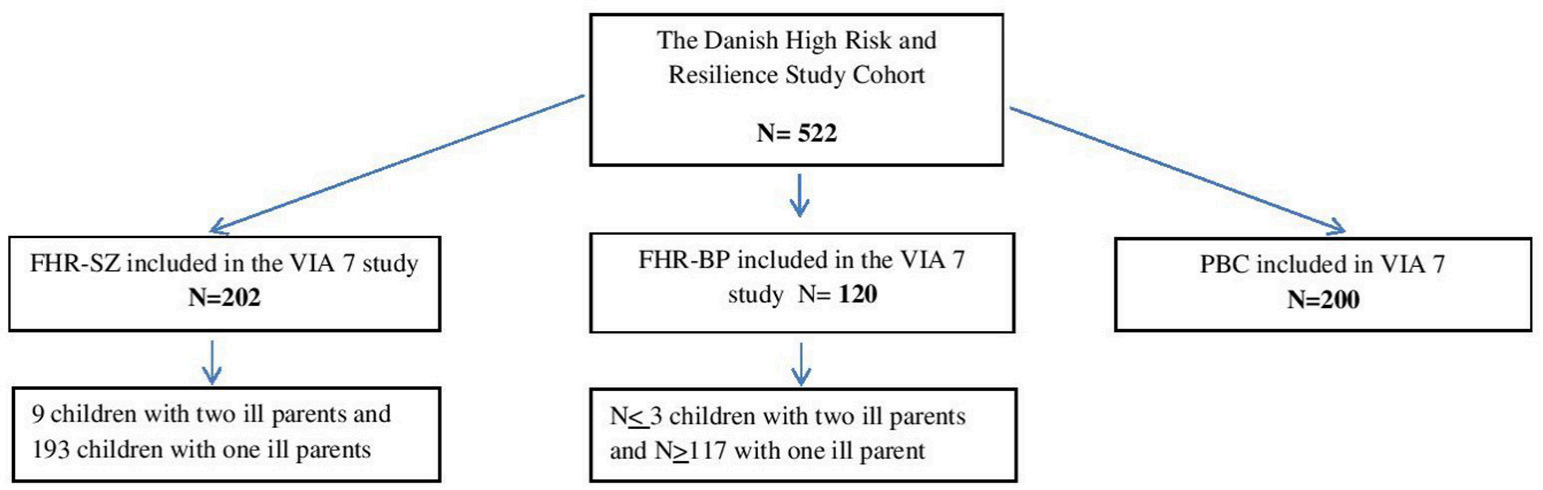
Flowchart of the Danish High Risk and Resilience Study illustrating all children being assessed in the VIA 7 study and who are now being assessed again.

The cohort consists of 522 individuals, now 11 years old (see Figure 1):

- 202 children with at least one parent diagnosed with schizophrenia spectrum psychosis
- 120 children with at least one parent diagnosed with bipolar disorder
- 200 children with neither of the parents treated in mental health services for the above diagnoses.

The children of parents with SZ and controls were matched on municipality, sex and exact age of the child. The representative cohort is based on data from The Danish Civil Registration System (27) linked to the Danish Psychiatric Central Research Register (28). Saliva from the children and blood samples from the parents were used for genome wide association analyses (GWAS, PsychChip). The children and their parents were thoroughly examined with interviews, neurocognitive, social cognitive and motor tests, questionnaires, home visits, and observations. Assessments were supplemented with data from questionnaires sent to school teachers. Outcomes for the children were assessed thoroughly in the domains of neuromotor functioning, neurocognitive, social cognitive functioning, social functioning, and psychopathology. Also, parents were interviewed about their mental health, and data on their neurocognitive as well as social cognitive functioning was collected.

Compared to previous studies this cohort is unique due to the very large representative sample of children, all of the same age at the time of assessment, which allows inference about development in the repeated cross-sectional examinations and ultimately it will allow us to follow developmental pathways in the longitudinal design. By including genetic material in combination with thorough estimates of environmental factors, measures of illness severity, and level of functioning of the parents we have a unique possibility of investigating the gene-environment interplay.

The full assessment battery in the VIA 7 study lasted ~3 days (5–6 h per day), as does the VIA 11 study-battery and the vast majority of the families completed the whole battery in the VIA 7 study. Parents were offered feedback on their child's performance, all participants received a gift card, and all families were informed that a follow-up at age 11 was planned.

## Methods

In the VIA 11 study we are aiming at re-assessing all children exactly 4 years from the date when they were included in the VIA 7 study. The adult who is taking care of the child on a regular basis and who is registered with the same address as the child is invited to give information (i.e., interviews and questionnaires) about the child's actual well-being and behavior (“primary caregiver”). This is often but not always one of the biological parents. The completion of the entire test battery lasts at least three days, and when organizing the appointments all kinds of flexible arrangements are being made to meet the families' specific needs without compromising the quality of the data collection.

All outcome measures are examined with validated instruments, specifically developed and selected for this age group, sensitive to small changes and suitable for later follow-up (Tables 1, 2). We will take advantage of the fact that most variables will be measured twice, making analyses of developmental predictors and associations possible between level of functioning at age seven and age 11, e.g., by multivariate analysis. Moreover, our analytic strategy will capitalize on the fact that the genetic exposures can be modeled directly with polygenic risk scores. Analyses will include measurement of the current environment to which the child is exposed. For each of the outcome domains listed below, we will analyse

1. the differences between the three groups of children at age 11,
2. the differences in associations of outcomes from age seven to age 11 in the three groups
3. the extent to which direct measures of environmental factors (e.g., home environment) and direct measures of genetic risk (e.g., polygenic risk scores for SZ and BD) are responsible for variations in outcomes and development.

**Table 1:** Domains and instruments used for testing the children at age 11 in the Danish High Risk and Resilience Study VIA 11.

**Domains**	**Outcomes**	**In VIA 7**	**Child**	**Primary caregiver**	**Teacher**	**Type of instrument**
Neuromotor and physical measures	Motor development and milestones	Yes	Movement ABC (29)			Test in clinic
	Anthropometry	Yes	Height, weight, waist			Observations in clinic
	Motor speed and dexterity	Yes	Finger tapping (30)			Test in clinic (or home)
	Physical activity and sleep	No	SENS chip (31)	Anamnestic interview		Chip on tight for one week (32) Interview with primary caregiver
Neurocognition	Verbal Memory and visual memory	Yes	Word Selective Reminding and Memory for Stories from Tomal-2 (33) RCFT (Rey Complex Figure Test and Recognition Trial (34) and Spatial Recognition Memory from CANTAB (35)			Test in clinic or at home
	Attention	Yes	RVP (Rapid Visual Information Processing;3-5-7 mode) from CANTAB (35). Conners CPT (Continuous Performance Test (36)			Computer test
	Communication and pragmatic/social interaction	Yes			CCC-2 (Children's Communication Checklist-II) (37)	Questionnaire
	Speed of Processing	Yes	Verbal Fluency 1-2 and Trail making Test 2-4 from D-KEFS (38) and Symbol Search, and Coding test from WISC-IV (39)			Test in clinic or home
	Executive functions (planning and flexibility)	Yes	SOC (Stockings of Cambridge) and IED (Intra-Extra Dimensional Set Shift) from CANTAB (35) and Verbal Fluency 3 from D- KEFS (38)			Computer test
	Executive functions (visual and verbal working memory)	Yes	SSP (Spatial Span) and SWM (Spatial Working Memory) from CANTAB (35) and Letter-number Sequencing and Arithmetic from WISC-IV (39)			Computer test and test in clinic or home
	Executive functions (error monitoring)	Yes	Flanker Task (40) – before and during fMRI			Computer test
	Social cognition	Yes No	Animated Triangles (41) before and during fMRI (without the goal directed animation)Frederic's stories (42)			Computer testPractical test
	Intelligence	Yes	RIST (Reynolds Intellectual Screening Test) (43)			Test in clinic or home
	Decision making	No Yes	Beads' Test (44)Cambridge			Computer test
			Gambling Task from CANTAB (35)		
Psychopathology	Psychiatric symptoms, incl. depression, anxiety, psychotic symptoms, thought disorders, PLEs, obsessive-compulsive symptoms, eating disorders, sleep disturbances, self harming behavior and traumatic life events	YesYesNoYes	Kiddie-SADS-PL interview (45)PLE assessment (46)Magical Ideation Questionnaire (47)	Kiddie-SADS-PL interview (45) Anamnestic interviewCBCL (48) (Child Behavior Checklist)	TRF (48) (Teachers Rating Form)	Interview (categorical psychopathology)Questionnaires, (dimensional psychopathology)
Do	Attention/hyperactivity	Yes		ADHD-Rating Scale (49)	ADHD-Rating Scale (49)	Questionnaire
	Affect regulation/flexibility	Yes	CEMS (50) (Children's Emotion	BRIEF (Behavior Rating	BRIEF (Behavior	Questionnaire
			Management Scale)	Inventory of Executive	Rating Inventory of	
				Function (51)	Executive Function (51)	
Do	Anxiety	Yes	STAIC (State-Trait Anxiety			Questionnaire
			Inventory for Children (52)			
Social functioning and behavior	Self-esteem	Yes	Sådan er jeg' ['I think I am' (53)]			Questionnaire
	Bullying	No	Olweus Bully/Victim			Questionnaire
			Questionnaire (54)			
			SSPS (State Social Paranoia Scale			Questionnaire after
			(55)			Virtual Reality scenario
	Resilience	No	CYRM (Child and Youth			
			Resilience Measure short version (56)			
	Social Functioning	Yes		SDQ (Strengths and Difficulties Questionnaire (57)	SDQ for teachers(Strengths and Difficulties Questionnaire (57)	Questionnaire (emotional symptoms, conduct problems, hyperactivity/ inattention)
		Yes			CCC-2 [Child Communication Checklist (37)]	
	Social development	Yes		Vineland AdaptiveBehavior Scales –II (58)		Interview
	Autism spectrum traits	Yes		SRS−2 (Social Responsiveness Scale (59)	SRS-2 (Social Responsive ness Scale (59)	Questionnaire
Environment and emotional climate	Stimulation and support in actual rearing environment	Yes	HOME Inventory, EarlyAdolescent version (60)	HOME inventory, Early Adolescent version (60)		Interview made in the home with both child and parent
	Life events and trauma	No	CTS [Childhood Trauma Screener (61)]	Anamnestic interview focusing on age 7-11		Questionnaire and semi-structured interview
	Perceived support from social network	Yes		SPS [Social Provision Scale (62)]		Questionnaire
	Attachment style	Yes, but new test	Secure Base Script Test (63)			Test in clinic or home
	Expressed emotions/emotional family climate/familiar relations	YesNo		FMSS [Five Minute Speech Sample (64)] FAD [Family Assessment Device (25)]		InterviewQuestionnaire
	Stress	Yes	Hair test for cortisol			Hair sample
			Items from DLSS [Daily Life			Questionnaire
			Stressor Scale (65)]			
	Use of social media	No		Anamnestic interview		Questionnaire
Physical health	Puberty status	No	Tanner stages (66, 67)			Illustrations
			Hormone level			Blood sample
	Physical health	No	HbA1c. leucocytes, CRP			Blood sample
			Exercise on bicycle,			Test (Copenhagen only)
Genetic and epigenetic analyses	Polygenic risk scores	Yes	Dry blood spots from Danish Neonatal Screening Biobank and blood samples			Day 1, hospital
	Inflammatory and infectious	Yes	Dry blood spots from Danish			Day 1, hospital
	markers.		Neonatal Scrbeening Biobank and			
			blood samples			
Brain scan	Functional and structural	No	MR Scanning and EEG			Day 2, Scan at hospital
	MRI					
	Electrophysiology	No	The Copenhagen			Day 2, before the scan at hospital
			Psychophysiological Test Battery			
			(CPTB) (prepulse inhibition, P50 gating, mismatch negativity)			

**Table 2:** Instruments for the assessment of the parents' mental health status, actual level of functioning and parenting issues in the Danish High Risk and Resilience Study - VIA 11.

**Domains**	**In VIA 7**	**Parent/actual caregiver**	**Co- parent**	**Type of test**
Mental health status (previous 4 years)	Yes (lifetime)	SCAN [Schedules for Clinical Assessment in Neuropsychiatry (68)] for the person him/herself and the other parent	do	Interview
Daily Functioning	Yes	PSP [Personal and Social Performance Scale (69)]	do	Interview
Actual state of illness	Yes	SANS [Scale for the Assessment of Negative Symptoms (70)]	do	Interview
Do	Yes	SAPS [Scale for the Assessment of Positive Symptoms (71)]	do	Interview
Do	Yes	Hamilton Rating Scale for Depression (72)	do	Interview
Do	Yes	YMRS [Young Mania Rating Scale (73)]	do	Interview
Affective regulation	Yes	ALS [Affective Liability Scale (74)]	do	
Family functioning	No	Family Assessment Device, short version [FAD-12 (25)]		Questionnaire
Perceived support from social network	Yes	Social Provision Scale [SPS (62)]	do	Questionnaire
Social Response, adult	No	Social Responsiveness Scale -Adult, self-report [SRS-A (75)]	do	Questionnaire
Relation to child and relation to other parent	Yes	FMSS [Five Minute Speech Sample (64)]		Interview (recorded and transcribed)
Adverse childhood experiences	No	ACE Study Questionnaire [Adverse Childhood Experiences Questionnaire (76)]	do	Questionnaire
Attachment style	Yes	PAM questionnaire (77)	do	Questionnaire
Knowledge about mental illness and talking to children about mental illness if relevant	No	Included in anamnesis		Interview

The analyses will thematically be divided into groups and will be carried out by Ph.D. students and postdoc researchers. Researchers are based in two centers, one in Copenhagen (Research Unit at Mental Health Center Copenhagen, Gentofte Hospital) and one in Aarhus (Psychosis Research Unit, Aarhus University Hospital Risskov) and have close collaboration at all levels including regular face-to-face meetings and video conferences. The outcomes are organized in the domains listed in Tables 1, 2 and described below.

## Domains and Instruments

### Neuromotor Function

Fine motor speed is being assessed with Finger Tapping Test (30), while manual dexterity, ball skills and balance are assessed with Movement Assessment Battery for Children-2, Movement ABC-2 (29). Physical activity will be measured by a sensor in an easily wearable adhesive patch [SENS motion® (31)] which can directly measure sleep disturbances and level of physical activity during a 1-week observation period.

### Neurocognitive Function

The comprehensive test battery includes Word Selective Reminding and Memory for Stories [TOMAL-2 (33)], Rey Complex Figure Test and Recognition Trial (34), Rapid Visual Information Processing [CANTAB (35)], Conners' CPT-II [Continuous Performance Test II (36)], Verbal Fluency 1–3 and Trail Making Test 2–4 [D-KEFS (38)], Symbol Search and Coding [WISC-IV (39)], Stockings of Cambridge, Intra-Extra Dimensional Shift, Spatial Recognition Memory, Spatial Span, and Spatial Working Memory [CANTAB (35)], Letter-Number Sequencing and Arithmetic [WISC-IV (39)], Cambridge Gambling Task [CANTAB (35)], and Reynolds Intellectual Screening Test [part of the RIAS (43)].

*Social cognition* is measured by Animated Triangles (41), Beeds Test (44) (tendency to jump to conclusion), Frederic's Stories (42), and the Social Cognition paradigm from the Human Connectome Project (78).

### Psychopathology

General psychopathology and psychotic-like experiences (PLEs) will be examined with the diagnostic interview K-SADS-PL (45) including a specialized assessment of sub-threshold psychotic-like experiences (79). Magical Thinking Questionnaire (47) is included to ensure optimal information about tendency to magical ideation that is not covered by the K-SADS-PL-interview.

The aberrant salience-hypothesis (80) suggests that patients with schizophrenia have deviances in attributional style. Results from the VIA 7 study showed that familial high-risk children more often reported being bullied (unpublished results), which could be a sad truth, but it could also be partly explained by deviances in attributional style. We have developed and included a predefined social situation in a virtual reality scenario that will help us identify any liability to social anxiety and paranoid ideas. By using a virtual reality scenario, we will be able to investigate if a subgroup of the children is more inclined than the others to appraise an ambiguous social situation as threatening or bullying using a virtual reality scenario. We will apply a Danish children's version of the State Social Paranoia Scale (55), which asks about positive, neutral and paranoid appraisal of the virtual reality environment.

Executive functioning including affective regulation and flexibility will be assessed with the questionnaire Behavior Rating Inventory of Executive Function [BRIEF (51)] from both caregiver and teacher. Autism spectrum traits are evaluated with Social Responsiveness Scale [SRS (59)] also completed by the child's caregiver and the teacher. Dimensional measures of psychopathology will be covered with Child Behavior Checklist [CBCL (48)], ADHD rating scale [ADHD-RS (49)], and Strengths and Difficulties Questionnaire [SDQ (57); both by parent and teacher] while the ratings of the clinical impression of the child during the testing are registered with the Test Observation Form [TOF (81)].

### Social Functioning and Behavior

Self-esteem will be coved by “I think I am”-questionnaire [Sådan er jeg (53)]. Social development is captured by the Vineland-2 Parental interview (58) and with the Strengths and Difficulties Questionnaire [SDQ (57)]. Data from the school will also be included via questionnaires sent to the teacher (see Table 1 for details). Bullying is included with the questionnaire developed by Olweus for the child itself (54). Resilience is measured by a short version of Child Youth Resilience Measurement [CYRM (56)] as well as by associating the life situation and context of the child with their course of psychopathology.

### Environment

Familial environment in the home is measured by the use of a semi-structured interview, HOME, that must take place at a home visit with the caregiver and the child being present at the same time [Home Observation for Measurement of the Environment (82)]. We chose the version called Early Adolescence (EA-HOME) which is appropriate for children from age 11. An anamnestic interview with the primary caregiver includes sociodemographic data, data on the child's health service use, adverse life events, school performance and leisure activities as well as social network. Childhood trauma is measured directly from the child through the section in the K-SADS-PL (45) about post-traumatic stress disorder (PTSD) combined with the Childhood Trauma Screener [CTS (61)]. Attachment style categorization will be based on Secure Base Script Test [SBST (63)] while level of stress will be captured by Daily Life Stressor Scale [DLS (65)] and from level of hair cortisol in a hair sample.

The parents' level of daily functioning is measured with Personal and Social Performance Scale [PSP (69)] and is used as a measure of the environment together with Five Minute Speech Sample (64), which contains data about the primary caregiver's impression of the relationship with the child.

### Physical Health Status

We will describe the children's physical condition including a long range of tests relevant for risk of physical illness at age 11. This will include: VO_2_max, anthropometry, Body Mass Index, white blood cells, C-reactive protein, HbA1c, and level of cortisol in hair samples. Puberty status will be determined by analyses of sex-hormones in blood tests and with the Tanner test (66, 67), which is letting the child look at drawings of different stages of puberty, and choose which stage is closest to the child's actual development.

### Genetic and Epigenetic Analyses

Saliva and peripheral blood are being sampled from the children and will be analyzed with DNA-analytic results from the dried blood spot from birth and from saliva-sample results collected at age seven with the purpose of studying epigenetic changes.

### Structural and Functional Magnetic Resonance Imaging (fMRI) an Electroencephalography (EEG)

We will examine the children with anatomical and functional magnetic resonance imaging (MRI) of the whole brain at 3.0 Tesla at Aarhus University, Center for Functionally Integrative Neuroscience (CFIN) and Hvidovre Hospital's, Danish Research Center for Magnetic Resonance (DRCMR). We will acquire 3D T1-weighted (MP2RAGE) and high-quality diffusion weighted imaging (DWI) data to calculate a set of measures that will allow us to detect regional changes in brain microstructure and structural brain connectivity (DWI based tractography) across groups. The measures include fractional anisotropy, apparent diffusion coefficient, and diffusion kurtosis indices. These measures will be used to compare regional changes in brain microstructure, as indexed by diffusion weighted imaging (DTI) across groups. Functional MR scans will map task related changes in brain activity and connectivity during three experimental tasks: the Eriksen Flanker paradigm, which addresses cognitive control and motivation, the Social Cognition (Animated Triangles) paradigm from the Human Connectome Project (78) which tap into theory of mind, and a Self-Reference paradigm to probe the ability to relate external events to oneself.

It is our intention to use these MR-scans carried out at age 11 as a basis for identifying developmental trajectories of brain changes during disease formation as we plan to repeat the same MR protocol later in adolescence (at age 15 or 16). The trajectory in children developing schizophrenia is expected to include reduced maturation of inhibitory pathways and excessive pruning of excitatory pathways leading to altered excitatory–inhibitory balance in the prefrontal cortex. We also anticipate structural and functional alterations of the brain's connectivity. Although some data support each of these possible neurodevelopmental mechanisms for schizophrenia, no study has previously provided possibilities for identifying measures of abnormal structural brain maturation (such as less or excessive pruning, less maturation of major limbic/cognitive fiber tracts, abnormal global brain connectivity, dysfunctional processing), and differences in integration in key circuits that will be probed with task-based fMRI and EEG in young individuals at risk for those disorders before puberty. We plan to correlate the abundant biological, clinical, and neurocognitive information already collected at age seven to future imaging data to see whether earlier data are predictive of imaging outcomes.

In a subgroup of ~50% of the participants, EEG will be performed at DRCMR to record functional task-related cortical activity during an auditory 40 Hz stimulation paradigm, a mismatch negativity paradigm, and a Flanker paradigm which is closely matched to the one used during functional MR to enable data integration. We selected these tasks based on consistent evidence for deficits in neural synchrony and event-related potentials (ERPs), evoked by using those paradigms in patients with SZ (83, 84) and, although to a lesser degree, in patients with BD (85). Reduced power of cortical activity and reduced amplitudes of event-related potentials in neural mechanisms are assumed to be related to both automatic sensory processes as to higher-order cognitive functions (86) and provide a window into some of the neurobehavioral symptoms (87). No study before has studied these neural correlates in a large sample of children at familial high risk for schizophrenia and bipolar disorder.

## Procedures and Permissions

### Contact and Procedures

All families have been informed by the completion of the first assessment in the VIA7 study that a follow-up was planned at age 11. To maintain contact with the participants of the cohort, Christmas cards and birthday cards have been sent to the children each year and a short survey consisting of two questionnaires [the “I think I am” (53) and the Child Behavior Check List CBCL (48)] was sent out to each family when the child turned 9 years of age; this survey is called the VIA 9 study.

**Figure 2:**
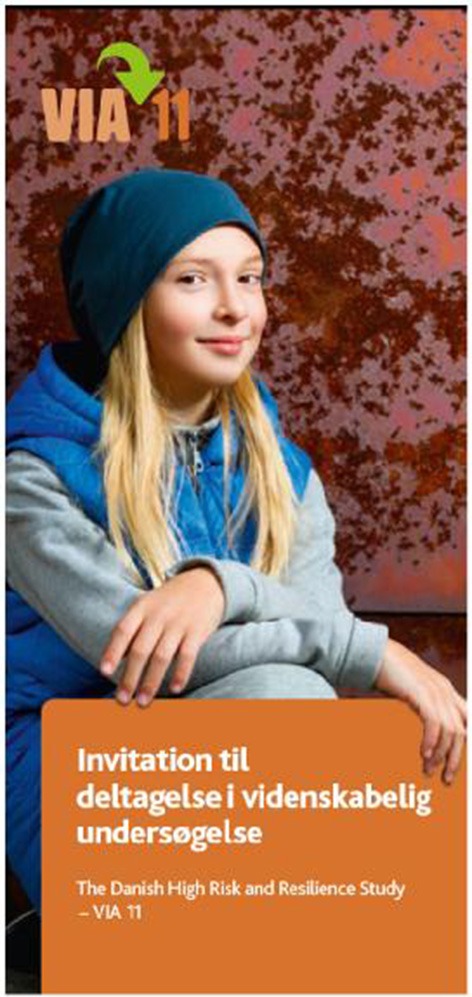
Lay out for the invitation to all families in the VIA 11 study.

As in the VIA 7 study, the first contact for the assessment is made by sending out a hard copy letter and an illustrative folder to the child's address (see Figure 2), followed by a phone call by the research coordinator, who invites the family to an information meeting. All parents give informed consent before data collection can start and for those who are divorced or separated and have joint custody over the child, informed consent from both parents is mandatory. We meet all families with a very friendly and flexible approach, taking into account any individual concerns or obstacles that they may have with regard to participation. The first results from the VIA 7 study are available for those participants who ask for them.

The data collection takes place during 3–4 meetings, each lasting up to ~5 h. The caregiver and the child are assessed simultaneously by two researchers, and whenever possible the assessor testing the child is kept blinded for the familial high-risk status and for the potential diagnosis from the first assessment at age seven to avoid any subjective influences. The first two days take place in the clinic, the third day is at the MR-scan facility, while the fourth day is in the family's home in order to complete the home environment assessment. However, some children may need more time or cannot concentrate sufficiently and thus need shorter but more frequent meetings. The child receives a gift card after each day completed and at the end of the whole assessment, the parents are offered a feedback on their child's performance and results. If any specific concern is raised on the basis of the interviews and testing, the researchers will guide the parents to relevant support or even make the referral or inform the social authorities or hospital service, if necessary. All diagnoses retrieved from the diagnostic interviews K-SADS-PL (45) and the SCAN interviews (68) are discussed and confirmed or declined at weekly clinical conferences, with a child and adolescent psychiatrist (A. Thorup) being present together with the research team members.

### Permissions and Data Protection

The study was approved by the local Ethical committee (Protocol number: H 16043682) and the Data Protection Agency (ID- number RHP-2017-003, I-suite no. 05333).

The majority of tests that we used in the VIA 7 study were chosen at the outset (among other reasons) because they would also be suitable at age 11 and this is a considerable advantage when aiming to study relatively small changes over time (Tables 1, 2).

### Statistical Analyses

Several descriptive and inferential statistical tools will be applied to analyse the data. For example, for descriptive analyses, frequency distribution, mean or median, and graphical representation will be applied. For inferential analysis, linear regression and/or analysis of variance or covariance (ANOVA/ANCOVA) will be the standard method for analyzing the association between exposure variables (i.e., high-risk status) and continuous outcome measures (e.g., psychopathology). If a variable does not follow a normal distribution even after possible transformations, non-parametric methods (e.g., Kruskal–Wallis test) or non-linear statistical models can be an alternative approach. Moreover, to control for the Type I error probability as well as the correlation between multiple outcome variables, multivariate analyses of variances/covariance (MANOVA/MANCOVA) will be applied. Furthermore, dichotomous/count outcome measures will be analyzed with generalized linear models (e.g., logistic or Poisson regression). To account for longitudinal effect from VIA 7 to VIA 11 data, linear mixed effect model for the continuous outcome and the generalized estimating equation for the dichotomous outcome will be applied. Based on our specific research question, we may consider clinically meaningful interaction terms (e.g., high-risk status and other covariates) in the models. Furthermore, as we are collecting genetic data for children and parents, to account for genetic background, polygenic risk scores (e.g., for Schizophrenia, education) would be considered as a covariate in the statistical models. In the case of missing data, multiple imputations will also be considered given that data are missing at random (MAR). However, in case of participating families with lower functioning there might be the chance that missing data are not at random (i.e., non-ignorable missing), but solely due to the inability or unwillingness to complete instruments, test, or questionnaires. Thus, we will apply sensitivity analyses for investigating the possible violations of the missing at random assumption. For families not participating at all, we will have the opportunity to include data from Danish registers in the sensitivity analyses. To handle the latent factors like resilience and vulnerability, we will apply structural equation modeling (SEM) or different kinds of path analyses. In addition, multiple regression will be applied for analyses of predictors and associations from age 7 to age 11 regarding changes in mental and physical symptoms.

## Results

Results will be presented within the context of both cross sectional and longitudinal analyses, i.e., comparing the results from the first assessment at age seven with the results found at age 11. Cross sectional results will be reported in all new areas or instruments like MR scans, Virtual Reality setting, SENS motion data, and blood sample data, while developmental trajectories will be investigated for those measures that are repeated, i.e., most of the cognitive measures, psychopathology, motor development, as well as many of the questionnaires, and the home environment evaluation (see Tables 1, 2).

Results concerning the actual psychopathology and the association to the mental health status at age seven will be investigated, including data on attributional style (tendency to report being excluded/bullied) and physical health. Data on social cognition will be linked to data on the developmental trajectories of Psychotic Like Experiences (PLE's; i.e., intensity, frequency, and severity) from age seven to age 11. Neurocognitive data will be analyzed to learn more about the extent to which these children's deficits or advantages measured at age seven remain stable, deteriorate or diminish over time. Brain scan data will focus on potential differences between high risk groups in terms of structural properties, neural networks, and connectivity.

## Discussion

This protocol describes the research strategy for the first follow-up of the unique familial high-risk cohort consisting of 522 children all the same age called The Danish High Risk and Resilience Study. This second wave is called the VIA 11 study since all children are now 11 years of age. The advantages of this cohort are especially that all children have the same age and that all families of the cohort have been recruited from national registries. Further, the comprehensive battery and the multidimensional approach will allow us to combine results from many research areas in order to add knowledge to the field of familial high risk, developmental psychopathology, brain development and risk, and resilience assessment.

The field of familial high risk studies is not new, actually the first study with this design was described and conducted by Barbara Fish (88) in New York in the early fifties, and from this study came the “pandysmaturation”-hypothesis, giving inspiration to the neurodevelopmental hypothesis of understanding the etiology of schizophrenia. In the field of schizophrenia research several familial high-risk studies have been conducted since Fish' study and recently reviewed by Hameed (89). However, we believe that the possibility of recruiting the participants from registries and not from clinics (i.e., biased toward including only those in treatment or with severe courses), with all children having the same age is a major advantage. From the perspective of neurodevelopmental psychopathology, age greatly matters when comparing results of cognitive tests, social and behavioral functioning and developmental milestones.

There are other important familial high-risk studies that are ongoing and should be mentioned here. One is the Canadian FORBOW study by Uher et al. (90) which is still recruiting both parents with schizophrenia, major depression and bipolar disorder and their offspring and testing various types of prevention and early interventions in the cohort, while also conducting annual assessments. The Spanish study led by Sanchez-Gistau (91, 92), called The Bipolar and Schizophrenia Young Offspring Study (BASYS), is a multi-center, naturalistic study which aims to evaluate psychopathology and neuropsychological and neuroimaging variables in child and adolescent offspring of patients with schizophrenia or bipolar disorder in two child and adolescent psychiatry departments in Madrid and Barcelona. The sample consists of 41 offspring of parents with schizophrenia, 90 offspring of parents with bipolar disorder and 107 controls. The age span is wide, 6–17 years and all participants are recruited via parents seeking treatment. The Dutch Bipolar Offspring Study led by Manon Hillegers in the Netherlands is a longitudinal fixed cohort study established in 1997 and followed up until early adulthood (93). It consists of 140 offspring from 86 families where one of the parents suffered from bipolar disorder. Many interesting results have been reported from this cohort, e.g., that the impact of life events especially in the early phases of the illness may be mediated by a passive reactive coping style. Further, a study led by Iacono et al. (94) has recent data on a smaller group of offspring of parents with bipolar disorder. However, all studies have much wider age ranges and smaller Ns.

### Conducting a Longitudinal Study

Conducting a longitudinal study and aiming for several assessments of both parents and children over time is an ambitious and demanding project. Longitudinal studies must be well-prepared and sufficiently financially supported and the team behind the study must set a long-term plan, have a strong collaboration and develop clear and good ways of communication with regular meetings and other forms of contact. Research questions must be based on a theoretical framework and reflect current state of the art and must map onto the aims of the study. Recruitment procedures of participants and strategies for retaining participants in the study are crucial and must be planned and discussed with potential participants/pilot users. The assessor group must be properly trained, have regular meetings and good collaboration, and high levels of interrater reliability is essential. Data must be handled in accordance with rules of data protection and analyzed on the basis of a statistical, preset strategy. Finally, results must be published for both professionals and the population (95). Communication of results that reveal some aspects that could be interpreted as negative for the participants must be planned and handled with empathy.

### Clinical Implications

Developmental trajectories will inform us on how the delays and abnormalities observed at age seven will evolve and influence the individual's risk of mental symptoms or reduced level of daily functioning at age 11. For example, much more knowledge is needed if we are aiming for understanding, treating or giving advice to children and their parents when children report psychotic like experiences (PLEs). Are PLEs at age seven just a normal, transient phenomenon that we as clinicians should not worry about? What is the impact if PLEs are found to correlate with an increased risk of psychopathology at age 11 and should persistent PLEs (i.e., found at both age seven and age 11) be considered as more severe early risk factor? We intend to investigate the nature of all possible PLEs mentioned by the children in the VIA 11 study in a very thorough way to be able to analyze data at a detailed level. Results from the VIA 7 study indicated that PLEs are much more common among FHR-SZ children (Ellersgaard et al, in preparation) and do correlate with poorer functioning, and this fact is important to follow very closely to help us learn more about their value as a risk marker for later mental illness, as proposed by others (96). A large cohort study reported that up to 2/3 of a population of school children aged nine to 11 would report some kind of PLEs on a self-report measurement, and for those individuals who reported the PLEs to be persistent after 2 years, a correlation with an increased risk for internal and external psychopathology later in life was found (96). However, methodological considerations are important, since other studies find much lower rates (97), for example the frequency was 5.9% based on a clinical interview in a large birth cohort of 12-year-old twins. In the VIA 11 study we initially considered using a self-report instrument, but concluded that due to the long test battery, the data collectors will have time to build a good relationship with the child and thus confidentiality will most likely be high, allowing the child to tell the interviewer if she or he has had any PLEs. Still, a well-validated rating scale for PLEs among children and adolescents with a clear and informative manual would be a considerable contribution to the field for the future. An adapted version of the SIPS/SOPS instrument (79) could be a place to start.

In a more general perspective, we hope that the results from this important cohort will shed light on the problems that these families face and struggle with every day. We appeal for authorities, institutions, and organizations, who are responsible for providing help and support to the whole population, to pay extra attention to families where severe mental disorders are involved, sometimes in two generations (see Box 1).

Box 1 **Case Benjamin**

Benjamin is a curious 11-year-old boy, who lives with his mother and his younger brother. Benjamin's mother has been diagnosed with bipolar disorder when Benjamin was four years old and she divorced Benjamin's father a few years ago. Benjamin visits his father every other weekend, sometimes more frequent, if his mother is depressed and in need of hospitalization. For most part of his life, Benjamin was told that his mother was hospitalized due to influenza, but recently Benjamin learned that it was because of her 'thoughts in her mind', that his mother sometime had to go away and letting him and his brother stay with their father and his new wife, and their baby twins.

Benjamin is usually a happy boy who likes to go to school, play football and Minecraft. However, he is also a rather anxious boy; who is not comfortable with sleeping at his friends or going too far away from home. He has also developed a sleep disorder as it takes him a very long time to fall asleep every night. At school and at home Benjamin sometimes gets very angry and has tantrums, although it is now much less frequent than when he was younger. He is doing fine in school and has one good friend. For a while, Benjamin was seriously bullied by some boys from another class. He was afraid to tell his mother about the bullying, because he wanted to protect her from getting sad. Eventually, the teacher found out and contacted Benjamin's mother. Now, the bulling has stopped but Benjamin still thinks a lot about having been bullied. Maybe that is why he sometimes have problems concentrating in school.

Benjamin participated in the VIA 7 study and the VIA 11 study and was fond of it, although the assessment at the hospital lasted three full days and was quite demanding. He talked to a psychologist in the VIA 11study about the sounds and voices that he had been hearing almost monthly for the past 8 months. The sounds and voices always seem to come when he is nervous or on his own, and although he realizes that they are not real, they make him feel uneasy. A few times he had shouted back at them. He had never talked to anybody about this before and found it as a relief to share it with a friendly person.

### Limitations

The comprehensive and thorough test battery is also quite time consuming which may lead to some families not being able to complete all instruments, tests, and questionnaires. Although we try to motivate every family to participate again, some degree of attrition is inevitable, and that this could be expected to be more pronounced in the group of lower functioning families, but it could also be in the busy and well-functioning ones. In the VIA 7 study, however, many families were positive about their participation and for some, the feedback on their child's performance and the results that we provided, meant a lot to them. We also offer all families a feed back after completed testing in the VIA 11, and in those cases, where the situation calls for immediate help or support from authorities, we try to guide the family, or we send referrals and information to the municipalities.

Since a large group of testers are working with the same instruments, we will be ensuring very carefully that all instruments are used, and results interpreted in the same way by having monthly meetings where we discuss any issues or problems and where we score clinical examples together. Testers often work together in pairs, but in varying combinations also with colleagues from the other center.

Most tests are chosen with the follow-up perspective, allowing us to compare results from the VIA 7 study to the VIA 11 study directly. However, this was not possible in some cases and it will thus be necessary to look into the comparability of tests. This is for example the case for the measure of attachment, where the method used in the VIA 7 study was the Story Stem Assessment Profile [SSAP (98)], which is suitable for age four to nine. In the VIA 11-study we use the Secure Base Script Test [SBST (63)], which is recommended from age 9 to 14. However, both tests are based on the same theoretical basis that a narrative setting and an invitation to create a story that is inspired from everyday situations will tap into the child's inner world and working models of adult- and self-presentations. Optimally, we would conduct a validity study with e.g., 30 nine-year-old children performing both tests and then see how the attachment profiles correspond in the two tests.

We decided to use two scanner facilities, one in Aarhus and one in Copenhagen to be able to cover all of Denmark apart from the similar scanner systems across sites, and to introduce the site variable in the data analyses, so that bias due the site will be detected. Finally, we will pay close attention to securing equal distribution of the scans of the cohort between the two centers in terms of HR-status of the children, so the two centers can analyze data independently.

Mean age of onset of puberty has lowered for the last decades (99), and for some children at age 11 the process of puberty has already started. We will be able to control for this difference in our analyses by using data from the blood tests and data regarding Tanner state of puberty as covariates.

### Perspectives

It is highly relevant to carry out careful examinations of early signs or symptoms of mental disorders among high-risk children several times before and during puberty to measure longitudinal patterns of symptoms. Especially, it is important to determine constellations and interactions with external influences that determine the event of a more negative progression of symptoms and function vs. constellations that facilitate resilience in an individual with a similar vulnerability. The current study allows us to explore these patterns, including a wide array of measures, reaching from phenotyping, to measures of brain structure and function.

The children at age 11 are facing puberty, which is a period characterized by massive changes in brain structures and connectivity as well as changes in physical appearance, hormonal status and psychological, and social constitution (13). Paradoxically, even though self-regulatory control develops at a fast pace during puberty, it is also a period with changes of behavior, including risk taking activities and new relational patterns, for example higher degrees of independency, and a period with high incidence rates for mental disorders. From a developmental perspective puberty is a period in life that is highly formative but also complex to study because both age, hormonal status and social and psychological aspects matter when comparing individuals in e.g., a cohort. This is why cohorts that focus on a narrow age range may be helpful in determining differences of development and behavior inter-individually, or systematically across specific groups. Social mechanisms, such as bullying or other forms of social defeat that may take place in childhood and adolescence, are frequently reported and are related to later emergence of mental illnesses like depression and psychosis (15) and other negative life outcomes.

It will be of immense value if a better understanding of the early phases of the development of mental disorders, neurodevelopmental changes including brain development and the needs of these children will permit early interventions with potential prevention or preemption of psychosis. Increased knowledge about the impact of the thoroughly described and closely investigated environmental risk factor like home environment, parenting style and relation, parental functioning and mental health, social network, socioeconomic status, and adverse life events will create the basis for developing specific supportive and preventive interventions. These children have for decades been known to be at an increased risk of mental illness. The more we know about the early development of their potential course of illness and resilience factors, the better we can provide early and specific help.

## Author Contributions

AET wrote the manuscript together with MN. All co-authors NH, AS, MG, ÅP, MK, JB, LC, MM, DE, BB, AG, MU, JO, AN, LJ, ALT, AA, LV, CK, HS, JJ, OM read the manuscript, commented etc. MU was responsible for the statistics section. KP, HRS, LØ, and VB were responsible for all content regarding the MR scans.

### Conflict of Interest Statement

The authors declare that the research was conducted in the absence of any commercial or financial relationships that could be construed as a potential conflict of interest.

## Abbreviations

ADHD: attention deficit and hyperactivity disorder
BASYS: The Bipolar and Schizophrenia Young Offspring Study
BD: bipolar disorder
DNA analytic: deoxyribonucleic acid
FHR: familial high risk
FHR-BP: familial high risk for bipolar disorder
FHR-SZ: familial high risk for schizophrenia
FORBOW (study): Families overcoming risks and building opportunities for well-being
GWAS: genome wide association analyses
MANOVA/MANCOVA: multivariate analyses of variances/covariance
MR/MR scan: magnetic resonance scanning
PLEs: psychotic like experiences
PTSD: post-traumatic stress disorder
SIPS/SOPS: Structured Interview for Prodromal Syndromes and Scale for Prodromal Symptoms
SZ: schizophrenia.
